# Parent-Child Diagnostic Agreement on Anxiety Symptoms with a Structured Diagnostic Interview for Mental Disorders in Children

**DOI:** 10.3389/fpsyg.2017.00404

**Published:** 2017-03-27

**Authors:** Lukka Popp, Murielle Neuschwander, Sandra Mannstadt, Tina In-Albon, Silvia Schneider

**Affiliations:** ^1^Clinical Child and Adolescent Psychology, Department of Psychology, Ruhr-Universität BochumBochum, Germany; ^2^Kinder- und Jugendpsychiatrische Klinik, Universitäre Psychiatrische KlinikenBasel, Switzerland; ^3^Clinical Child and Adolescent Psychology and Psychotherapy, University of Koblenz-LandauLandau, Germany

**Keywords:** parent-child agreement, parental psychopathology, structured diagnostic interview, kinder-DIPS, anxiety disorders

## Abstract

**Objective:** In clinical structured diagnostic interviews, diagnoses based on parent and child reports have low to moderate agreement. The aims of the present study are (1) to examine diagnostic agreement on anxiety disorders between parents and children on the levels of current and lifetime diagnostic category and diagnoses focusing in particular on diagnostic criteria and (2) to identify parent- and child-related predictors for diagnostic agreement.

**Method:** The sample consisted of 166 parent-child dyads interviewed with the Structured Diagnostic Interview for Mental Disorders in Children (Kinder-DIPS, Schneider et al., 2009). The children (51.8% girls) were between the ages of 7 and 18 years (*M* = 10.94; *SD* = 2.22).

**Results:** Overall, parent-child agreement on the diagnostic category of anxiety disorder (*k* = 0.21; *k* = 0.22) and the specific anxiety diagnoses (base rate > 10%) of social phobia, specific phobia and separation anxiety disorder (*k* = 0.24–0.52; *k* = 0.19–0.43) and corresponding diagnostic criteria (*k* = 0.22–0.67; *k* = 0.24–0.41) were low to moderate with the highest agreement on separation anxiety disorder (*k* > 0.43). Lower maternal depression, and higher social support reported by mother and father were associated with higher parent-child agreement. Maternal depression was indicated as the strongest predictor. Parental sense of competence, parental anxiety, the amount of parent-child interaction and the child's age and gender had no predictive value.

**Conclusions:** Parent-child agreement can be expected to be higher on the level of anxiety criteria compared to specific anxiety diagnoses and diagnostic anxiety category. Psychological strains in the family—especially maternal depression and low social support—lower the parent-child agreement on anxiety symptoms. Child- and relation-related variables (age, gender, amount of time parent(s) and children interact) play no role in the prediction of low parent-child agreement.

## Introduction

Anxiety disorders are the most common mental health disorders in children and adolescents (collectively “children”) with a worldwide-pooled prevalence of 6.5% (Polanczyk et al., 2015). The reliable and valid identification of anxiety disorders is an important clinical task, as anxiety disorders in childhood are a major risk factor for the development of further mental disorders (e.g., Woodward and Fergusson, 2001; Kossowsky et al., 2013). Structured (including semi-structured) interviews are the “gold standard” for diagnosing mental disorders (Costello et al., 2005; In-Albon et al., 2011; Merten and Schneider, in press). Interviews permit exchange between interviewer and interviewed person and require less literacy in the interviewed person, among other advantages. The assessment of child psychopathology requires diagnostic information from multiple informants to increase the validity and reliability of the assessment (Achenbach, 2006). Although, studies have shown that children and adolescents report their own emotional or behavioral problems reliably and validly (Arseneault et al., 2005; Luby et al., 2007), parents contribute valuable information of a different kind about the child's behavior and emotional problems (Achenbach, 2006).

An early benchmarking meta-analysis on cross-informant agreement about behavioral and emotional problems of 1.5–19-year-old children by Achenbach et al. (1987) yielded low mean kappa values of about 0.25 for the agreement between child and parent on diagnostic information. Higher but still low agreement (*k* < 0.40) was reported in recent studies for anxiety disorders (e.g., Jensen et al., 1999; Grills and Ollendick, 2003; Choudhury et al., 2003) with the highest agreement for separation anxiety disorder (SAD) (*k* < 0.32), followed by social phobia (SoP) (*k* < 0.28) and specific phobia (SP) (*k* < 0.24; Grills and Ollendick, 2002; Choudhury et al., 2003; Brown-Jacobsen et al., 2011). Overall, parents were found to report more diagnoses of anxiety than their children. However, more diverse findings were found about the distribution of endorsed criteria between parents and children according to separate specific anxiety disorders. For example, children reported more criteria of SAD whereas parents endorsed more criteria of SoP, SP, and GAD (Choudhury et al., 2003; Reuterskiöld et al., 2008).

Earlier studies showed that parents and children agreed more on diagnostic problems that are concrete, observable, severe, and unambiguous (Herjanic and Reich, 1982; Salbach-Andrae et al., 2009; Hoffman and Chu, 2015). Comer and Kendall (2004), for example, found higher agreement on symptoms compared to diagnoses assessed with the Anxiety Disorders Interview Schedule (ADIS; Silverman and Albano, 1996). Additionally, they found higher agreement on diagnostic symptoms that were observable and not school-related.

According to the Kinder-DIPS, a low to moderate agreement (average *k* = 0.26) across all diagnostic criteria were found in a community sample (*N* = 234, age range 11–17) (Propp et al., 2014). Parent-child agreement could not be reported on the level of specific diagnoses due to low base rates (0–4%). Therefore, no explicit results for diagnostic levels of anxiety disorders were reported.

## Predictors of parent-child agreement

Several reviews showed that informant characteristics influence parent-child agreement (Reyes and Kazdin, 2005; Smith, 2007). The present results substantiate and add to previous findings, as, in contrast to earlier studies, both informant- and relation-related variables specified for anxiety disorders will be investigated. Informant-related predictors such as the child's age and gender as well as parental psychopathology such as depression and anxiety have been frequently studied. Relation-related variables such as the amount of parent-child contact, social support or parental sense of competence have been rarely investigated. The following section provides the current state of research for these variables.

Several studies examined the predictive value of the child's age and gender on parent-child diagnostic agreement with inconsistent results. Indications for a higher parent-child agreement was found for older and younger age groups as well as for boys and for girls (e.g., Edelbrock et al., 1986; Achenbach et al., 1987; Rapee et al., 1994; Jensen et al., 1999; Sourander et al., 1999; Berg-Nielsen et al., 2003; van der Ende and Verhulst, 2005). The majority of studies found no influence of age or gender (e.g., Cantwell et al., 1997; Choudhury et al., 2003; Grills and Ollendick, 2003; Martin et al., 2004; Karver, 2006; Kiss et al., 2007).

Anxious or depressed parents reported more clinical symptoms in their children than parents without psychopathological problems (Briggs-Gowan et al., 1996; Chilcoat and Breslau, 1997; Najman et al., 2000; Chi and Hinshaw, 2002; Treutler and Epkins, 2003; Rothen et al., 2009). Two different hypotheses are salient about the interpretation of the report of anxious or depressed parents. The first hypothesis is that parents overestimate the amount of symptoms due to their own negative bias (Richters, 1992). The second hypothesis describes that depressed parents are more accurate in evaluating their children's anxiety- or depression-related symptomatology. This is supported by the depression-realism effect that indicates that depressed people make more accurate and realistic judgments than non-depressed people, the so-called “sadder but wiser” phenomenon (Alloy and Abramson, 1979; Moore and Fresco, 2012).

Some researchers suggest that agreement is less influenced by age and gender than by the total time parents and children spend together (Grills and Ollendick, 2003; Treutler and Epkins, 2003). It is expected that parents who spend more time with their children are presumed to know more about the emotions and behaviors of their children than parents who spend less time with their offsprings. However, no empirical data are available answering this research question.

Parents with a high level of social support and self-efficacy showed increased parental capacity including responsive caretaking and higher attention to the child's signals (Coleman and Karraker, 2000). It has been shown that if parental capacity is reduced by depressive symptomatology, parents feel more competent in their parental role if they have a social supportive environment (Kötter et al., 2011). Thus, both factors are expected to lead to higher parent-child agreement.

In sum, parent-child agreement was found to be generally low on structured diagnostic interviews. Agreement on anxiety disorders was found to be higher than in other childhood disorders. However, variances in agreement were indicated according to the diagnostic level, type of disorder, and characteristics of criteria (e.g., observability). Furthermore, different informant- and relation-related predictors were identified to influence parent-child agreement. Until now, the impact of type of diagnostic levels on parent-child agreement in childhood anxiety and possible predictors like parental self-efficacy and quantity of parent-child interaction were not investigated for structured diagnostic interviews in a clinical and non-clinical sample. Therefore, the aims of the present study were to investigate (1) the amount of parent-child agreement on three different diagnostic levels (diagnostic category, diagnoses, and diagnostic criteria) of childhood anxiety disorders using the Kinder-DIPS and (2) the predicting value of informant- and relation-related variables on parent-child agreement on diagnostic criteria. Based on previous research, we hypothesized that parents and children agree low on the diagnostic category and diagnoses but moderate on diagnostic criteria. We hypothesized, furthermore, that diagnostic agreement is higher between parents who spend more time with their younger children compared with parents and older children. In line with earlier studies, parent-child agreement is expected to decrease when parental depression and anxiety increases and social support and parental sense of competence decreases.

## Materials and methods

### Participants

A total of 257 Kinder-DIPS interviews were conducted at either the outpatient clinic (54.5%) of the University of Basel or at psychiatric and psychological services (45.5%) in Basel. Participants interviewed at the two sites did not differ considerably in terms of demographic information: Children in both groups were around 11 years old (*M* = 10.63–11.95, *SD* = 1.63–3.52) and parents were in their early forties (*M* = 41.68–45.14, *SD* = 4.73–6.30). The group seen at the outpatient setting (University) included 44.6% boys while of the group interviewed at the psychiatric and psychological services 55% were boys. A greater number of parents seen at the outpatient setting (82%) were married compared to parents at the psychiatric and psychological services (60%).

Demographic information largely matched when the interview partner was the mother or both parents: The participants were similar with respect to children's age (*M* = 10.89–11.01); *SD* = 2.00–2.54) and gender (boys = 47.1–50.0%). Also, in both groups, mothers respectively mother and father were in their early forties (*M* = 42.4–44.70, *SD* = 4.79–5.48). There was a difference between the two groups with respect to the marital status. 68.8% of mothers interviewed alone were married, whereas 81.4% of parents interviewed together were married.

In 147 (57.2%) interviews a diagnosis of at least one anxiety disorder as primary diagnosis was assigned. In 32 parent-child dyads no diagnosis (12.5%) was assigned. In additional 78 (30.4%) interviews another clinical disorder except anxiety disorders was assigned, however, these interviews were excluded from our analyses to maintain the focus on anxiety disorders. Interview partners were mothers (36.3%), fathers (1.7%), or both parents (56.4%). Interview data with fathers and missing information about the interview partner (5.1%) were deleted pairwise from further analyses.

The final sample consisted of 166 children (51.8% girls) between the ages of 6 and 18 years (*M* = 10.94; *SD* = 2.22). Of them 128 (77.1%) were interviewed in the outpatient clinic of the University of Basel and 38 (22.9%) at psychiatric and psychological services. Mothers' and fathers' average ages were 41.81 (*SD* = 4.84) and 45.13 (*SD* = 6.89), respectively (for an overview of sociodemographic data see Appendix A). A subgroup of participants (*n* = 129) filled out questionnaires to investigate parent and child related predictors on parent-child agreement.

### Participant recruitment and selection procedures

Participants were recruited for a treatment study for SAD at the University of Basel and from psychiatric and psychological services (Kinder- und Jugendpsychiatrie Baselland, KJP; Schulpsychologischer Dienst Basel-Stadt, SPD) between May 2004 and September 2008. Participants recruited at the University of Basel were screened for anxiety disorders before they underwent the Kinder-DIPS while participants recruited at psychiatric and psychological services were not selected by a specific primary diagnosis. The Kinder-DIPS was part of the initial diagnostic procedure. All participants needed a basic level of German literacy to complete the diagnostic assessment. Exclusion criteria for all participants were a comorbid diagnosis of pervasive developmental disorder and acute drug abuse.

### Procedure

The studies had been approved by the local ethical committee for medical research. Prior to the interview, children and parents were informed about the interview process and gave their written informed consent. For children younger than 16 years old, parents gave their written informed consent. Parents and children were interviewed separately in random order, having different interviewers for their Kinder-DIPS interviews. For each interview current and lifetime diagnoses were derived. Interviewers were blinded to the child's referral reasons and they conducted parent and child interviews independently. All interviews were recorded and subsequently recoded by different raters to ensure interrater reliability. There were three diagnostic profiles for each child-parent(s) couple: (a) clinical diagnoses according to the child's interview, (b) clinical diagnoses according to the parents' interview, (c) a composite rating between a and b. The inter-rater reliability was calculated for the child and the parent interview separately. The diagnosis with the most severe rating (child or parent interview) was labelled as the primary diagnosis. Discrepant clinical ratings were discussed during regularly held conferences with two senior clinical supervisors until consensus was reached.

Prior to the interview, parents and children filled out several questionnaires which are described in detail in the measures section. Additionally, parents indicated how many hours per week they usually spend with their children.

### Measures child

#### The structured diagnostic interview for mental disorders in children (Kinder-DIPS), original German version (2nd edition).

The Kinder-DIPS (Schneider et al., 2009) is a DSM-IV-TR-based (American Psychiatric Association, 2000) structured diagnostic interview assessing the most important areas of child psychopathology, as well as basic sociodemographic variables, in youths aged 6–18 years. The interview comprises independent, largely parallel parent and child versions. Inter-rater reliability for parent and child interviews in outpatient and inpatient settings is reported as good to very good for lifetime and current major diagnostic categories and diagnoses (Cohens Kappa 0.88–0.98; Neuschwander et al., 2013).

### Measures parents

The following questionnaires were filled in by the mother and the father of the child.

#### Beck depression inventory (BDI), German version.

Parents completed the BDI (Hautzinger et al., 1994), a widely used self-administered measure for the assessment of dysphoria and clinical depression, with high internal consistency (α = 0.88 for clinical samples) and good test-retest reliability (Hautzinger, 1991). In the present sample, the internal consistency was α = 0.79 for mothers' and α = 0.78 for fathers' reports.

#### Beck anxiety inventory (BAI), German version

Parents filled in the Beck Anxiety Inventory (Beck and Steer, 1993; German translation: Margraf and Ehlers, 2002) a 21-item self-report questionnaire assessing current cognitive and somatic symptoms of anxiety on a 4-point scale. A high internal consistency (α ≥ 0.90 for clinical, α ≥ 0.75 for non-clinical samples), test-retest reliability (*r* ≥ 0.68) and validity with different anxiety scales (*r* ≥ 0.46) are reported (Goldschmidt and Berth, 2008). Internal consistencies of the parents' report were obtained in the present sample (mother: α = 0.90, father: α = 0.89).

#### Anxiety sensitivity index (ASI), German version

The ASI (Reiss et al., 1986; German translation: Ehlers, 1986) is a self-administered 16-item list that measures anxiety sensitivity, the fear of anxiety and anxiety-related mental and bodily sensations on a 5-point Likert scale. High internal consistency (α > 0.80), and stable test-retest reliability (*r* > 0.70) are reported (Peterson and Plehn, 1999). Internal consistency in the present sample was α = 0.83 for both mothers' and fathers' reports.

#### Parenting sense of competence (PSOC) scale, German version

Parents filled in a 16-item self-report questionnaire assessing the perceived sense of parental competence on a 6-point Likert scale (Johnston and Mash, 1989; German translation: Miller, unpublished dissertation) on two scales “satisfaction” and “self-efficacy.” For the present purposes, the scale was reversed so that higher values indicated a higher amount of perceived parental competence. A high internal consistency (α ≥ 0.70) has been reported for the two subscales and the total scale (Johnston and Mash, 1989; Gilmore and Cuskelly, 2008) that was replicated for both parents in the present sample with α = 0.80.

#### Social network and social support

Parents filled in two separate 10-item sets of questions measuring how much social support they receive and how large their social support network is on 4-point scales, translated and adapted version from The “Avon Longitudinal Study of Parents and Children” (ALSPAC) (O'Connor et al., 1999). Internal consistency in the present sample was for mothers' α = 0.77 and for fathers' reports α = 0.85.

### Interviewers

Forty-eight interviewers (39 psychologists with Bachelor's degree; 9 psychologists with Master's degree), with a standardized training in the administration and scoring of the Kinder-DIPS conducted the interviews (see Kinder-DIPS manual for DSM-IV-TR; Neuschwander et al., 2013). All clinicians successfully completed a certification process under supervision. High inter-rater reliability for the parent (α ≥ 0.88) and child version (α ≥ 0.92) in the Kinder-DIPS for anxiety disorders were reported (Neuschwander et al., 2013).

### Data analyses

Agreement between children and parents was computed using correlational statistics with SPSS 22.0.0.0 for Mac. All agreement statistics were calculated within both a “current” and a “lifetime” frame, as well as on three diagnostic levels: (1) diagnostic category (presence or absence of an anxiety disorder), (2) diagnoses (SAD, SoP, SP), and (3) diagnostic criteria. Kappa coefficient (*k*, Cohen, 1960) and Yule's Y coefficient (Yule, 1912) were calculated to examine the parent-child agreement. The received values of kappa coefficient < 0.4 indicates low agreement, 0.4–0.6 moderate, 0.6–0.8 good, and >0.8 excellent agreement (Fleiss, 1981). Statistical significance of the kappa coefficient was determined with *x*^2^-exact tests. The Yule (1912) coefficient was proposed as a chance-corrected measure of agreement that is, contrary to the kappa statistic, independent of the prevalence of the observed event (Spitznagel and Helzer, 1985). Based on the odds ratio (standardized to range from −1 to 1), the Yule's Y is comparable to other correlational coefficients (Wirtz and Caspar, 2002). The kappa and Yule's Y statistics reduce reliability if prevalence of the observed events is low resulting in symmetrically or asymmetrically skewed marginal distributions of the contingency tables (Kraemer, 1979; Maclure and Willett, 1987; Thompson and Walter, 1988; Cicchetti and Feinstein, 1990; Feinstein and Cicchetti, 1990). Correlational statistics of parent-child agreement were only reported for diagnoses with a higher base-rate than 10% (a complete table of inter-rater measurements is available upon request). Additionally, total agreement as the percentage of the number of agreement between parent and child were reported.

For the investigation of child- and parent related predictors of diagnostic agreement, the frequency of disagreements rather than agreements on the diagnostic criteria per parent-child dyad were calculated to minimize the chance agreement on the absence of symptoms. Chance agreement on the absence of symptoms is typically high since children report a relative limited number of positive diagnostic criteria.

The predictors of parent-child agreement were investigated in three steps. First, unadjusted bivariate correlations (Pearson's *r*, two-tailed) were calculated between the number of parent-child agreement and predictor variables. Second, if there was a significant correlation between a predictor variable and the number of disagreement, a linear regression analyses and finally a multiple regression analyses were conducted with these variables. Child related variables were gender, age, and the amount of interaction time between child and parents. Parent related variables were scores on psychopathological (BDI, ASI, BAI), parenting (PSOC), and social support questionnaires.

## Results

### Parent-child agreement

According to parental report, 113 (68.1%) of the children met DSM-IV-TR criteria for a current anxiety disorder, and 122 (71.3%) of the children met DSM-IV-TR criteria for any lifetime diagnosis. According to child report, 79 (47.6%) of the children met DSM-IV-TR criteria for any current diagnosis, and 89 (52.0%) of the children met DSM-IV-TR criteria for a lifetime anxiety disorder.

Overall, children and parents agreed significant, but low to moderate on current and lifetime diagnostic information (see Tables 1, 2). On the level of diagnostic category, kappa values about the presence of a current (*k* = 0.22) or a lifetime (*k* = 0.21) anxiety disorder were low. On the level of current and lifetime diagnoses, kappa values (base rate > 10%) were low to moderate for SAD (*k* = 0.52; *k* = 0.43), SP (*k* = 0.24; *k* = 0.19), and SoP (*k* = 0.25; *k* = 0.28), indicating the highest agreement for SAD (current and lifetime diagnoses). Contingency tables (Table 1) showed that parents reported more current diagnoses than their children across all current and lifetime anxiety disorders.

**Table 1:** **Parent-child agreement on current and lifetime DSM-IV-TR diagnostic anxiety symptoms on the levels of the diagnostic category and diagnoses assessed with the Kinder-DIPS**.

	**Frequencies (parent/child)**		**Estimated prevalence (%)**	**Total agreement**	**Cohen's kappa (95% CI)**	**Yule's Y**
	**−/−**	**−/+**				
	**+/−**	**+/+**				
**CURRENT**						
Anxiety	36	15	94.5 (57.6%)	60.0%	0.22^*^ (0.05–0.35)	0.27
Disorders	50	62				
**LIFETIME**						
Anxiety	30	16	101 (62.4%)	61.7%	0.21^**^ (0.07–0.35)	0.26
Disorders	46	70				
**CURRENT DIAGNOSES**						
Separation anxiety disorder (309.21)	96	7	52 (31.5%)	79.4%	0.52^***^ (0.40–0.66)	0.62
	27	35				
Specific phobia (300.29)	110	15	29.5 (18.6%)	75.5%	0.24^**^ (0.02–0.37)	0.27
	24	10				
Social phobia (300.23)	120	7	25.5 (15.7%)	79.6%	0.25^**^ (0.07–0.43)	0.42
	26	9				
No diagnosis	62	50	68.5 (42%)	60.1%	0.22^**^ (0.08–0.35)	0.27
	15	36				
**LIFETIME DIAGNOSES**						
Separation anxiety disorder (309.21)	89	11	58.5 (34.4%)	73.5%	0.43^***^ (0.29–0.56)	0.49
	34	36				
Specific phobia (300.29)	113	16	32 (19.3%)	74.7%	0.19^*^ (0.02–0.36)	−0.25
	26	11				
Social phobia (300.23)	122	7	28.5 (17.0%)	79.2%	0.28^**^ (0.11–0.45)	0.45
	28	11				
No diagnosis	70	46	61 (37.7%)	61.7%	0.21^**^ (00.07–0.35)	0.26
	16	30				

*Sample size for diagnostic categories vary due to missing values. Kappa coefficients are provided for diagnoses with a base rate higher than 10%. Kappa coefficients are not calculated if no disorder is identified by at least one informant. Yule's Y coefficients are incalculable if either cell frequency of the contingency tables equals zero. Significance of the kappa coefficients was determined with x^2^-exact tests*.

* p < 0.05;

** p < 0.01;

*** *p < 0.001*.

**Table 2:** **Parent-child agreement on current and lifetime DSM-IV-TR anxiety symptoms on the level of diagnostic criteria assessed with the Kinder-DIPS**.

	**Frequencies (parent/child)**		**Estimated prevalence (%)**	**Total agreement**	**Cohen's kappa (95% CI)**	**Yule's Y**
	**−/−**	**−/+**				
	**+/−**	**+/+**				
**CURRENT SEPARATION ANXIETY DISORDER**						
A: Developmentally inappropriate and excessive anxiety concerning separation from home or from caregivers	90	8	56	79.1	0.53^***^	
	26	39	(34.4)		(0.41–0.68)	0.61
B: Duration of symptoms at least 4 weeks	80	10	68	82.7	0.65^***^	
	18	54	(42.0)		(0.53–0.76)	0.66
C: Onset before age of 6 years	80	10	69	84.0	0.67^***^	
	16	56	(42.6)		(0.56-0.79)	0.68
D: Clinically significant distress or impairment in social, academic, or other important areas of functioning	89	8	59	61.5	0.63^***^	
	20	45	(27.1)		(0.51–0.75)	0.67
E: Symptoms are not better accounted for by another mental disorder, i.e., panic disorder with agoraphobia	81	10	68	62.4	0.67^***^	
	16	55	(31.2)		(0.56–0.79)	0.68
**CURRENT SPECIFIC PHOBIA**						
A: Marked and persistent, excessive fear that is cued by the presence or anticipation of a specific object or situation	68	26	67	68.8	0.63^***^	
	24	42	(41.9)		(0.21–0.51)	0.36
B: Exposure to the phobic stimulus almost invariably provokes an immediate anxiety response, which may take the form of a panic attack	77	18	60.5	71.9	0.40^***^	
	27	38	(37.8)		(0.26–0.55)	0.42
C: The person recognizes that the fear is excessive or unreasonable. In children, this feature may be absent	96	24	40	73.2	0.30^**^	
	18	19	(25.5)		(0.13–0.46)	0.35
D: The phobic situation is avoided or else endured with intense anxiety or distress	68	25	67	68.8	0.36^***^	
	25	42	(41.9)		(0.21–0.51)	0.36
E: Symptoms interfere with the person's normal routine, or cause marked distress	100	16	38.5	73.1	0.27^**^	
	27	17	(24.1)		(0.10–0.43)	0.33
F: Duration of at least 6 months in individuals under 18 years	70	25	64.5	68.1	0.34^***^	
	26	39	(40.3)		(0.19–0.49)	0.34
G: Symptoms are not better accounted for by another mental disorder	103	28	72	72.4	0.40^**^	
	28	44	(35.5)		(0.27–0.53)	0.41
**CURRENT SOCIAL PHOBIA**						
A: A marked and persistent fear of one or more social or performance situations	105	11	35	73.9	0.25^**^	
	31	14	(21.7)		(0.09–0.41)	0.35
B: Exposure to the feared social situation almost invariably provokes anxiety, which may take the form of a panic attack	104	31	37.5	75.8	0.34^***^	
	8	18	(23.3)		(0.18–0.50)	0.47
C: The person recognizes that the fear is excessive or unreasonable. In children, this feature may be absent	115	15	23	82.8	0.33^***^	
	11	10	(15.2)		(0.13–0.53)	0.45
D: The phobic situation is avoided or else endured with intense anxiety or distress	105	9	38.5	77.2	0.38^***^	
	28	20	(23.8)		(0.22–0.54)	0.49
E: Symptoms interfere with the person's normal routine, or cause marked distress	113	7	29.5	75.9	0.22^**^	
	32	10	(18.2)		(0.06–0.38)	0.38
F: Duration of at least 6 months in individuals under 18 years	96	11	43	72.7	0.32^***^	
	33	21	(26.7)		(0.17–0.47)	0.40
G: Symptoms are not due to the direct physiological effects of a substance or a medical condition	97	11	42	72.7	0.31^***^	
	33	20	(26.1)		(0.16–0.46)	0.40
H: Symptoms are unrelated to or another mental disorder or medical condition	98	10	42	73.9	0.34^***^	
	32	21	(26.1)		(0.19–0.49)	0.43
**LIFETIME SEPARATION ANXIETY DISORDER**						
A: Developmentally inappropriate and excessive anxiety concerning separation from home or from caregivers	76	9	62.5	75.3	0.49^***^	
	30	43	(39.6)		(0.36–0.63)	0.55
B: Duration of symptoms at least 4 weeks	64	13	74	75.8	0.52^***^	
	25	55	(47.1)		(0.39–0.65)	0.53
C: Onset before age of 6 years	64	14	76	77.2	0.55^***^	
	22	58	(48.1)		(0.42–0.67)	0.55
D: Clinically significant distress or impairment in social, academic, or other important areas of functioning	78	9	62.5	79.0	0.57^***^	
	24	46	(39.8)		(0.44–0.70)	0.61
E: Symptoms are not better accounted for by another mental disorder, i.e., panic disorder with agoraphobia	66	13	74.5	77.8	0.56^***^	
	22	57	(47.2)		(0.43–0.69)	0.57
**LIFETIME SPECIFIC PHOBIA**						
A: Marked and persistent, excessive fear that is cued by the presence or anticipation of a specific object or situation	64	24	64	70.7	0.40^***^	
	20	42	(42.7)		(0.21–0.51)	0.41
B: Exposure to the phobic stimulus almost invariably provokes an immediate anxiety response, which may take the form of a panic attack	68	19	60.5	70.2	0.38^***^	
	26	38	(40.1)		(0.23–0.53)	0.39
C: The person recognizes that the fear is excessive or unreasonable. In children, this feature may be absent	84	24	38	71.8	0.28^**^	
	16	18	(26.8)		(0.11–0.46)	0.33
D: The phobic situation is avoided or else endured with intense anxiety or distress	64	23	63	70.5	0.40^***^	
	21	41	(42.3)		(0.25–0.54)	0.40
E: Symptoms interfere with the person's normal routine, or cause marked distress	88	17	37	71.2	0.24^**^	
	25	16	(25.3)		(0.07–0.42)	0.29
F: Duration of at least 6 months in individuals under 18 years	64	24	64.5	68.0	0.34^***^	
	25	40	(42.1)		(0.19–0.49)	0.35
G: Symptoms are not better accounted for by another mental disorder	65	24	61	71.4	0.41^***^	
	18	40	(41.5)		(0.26–0.56)	0.42
**LIFETIME SOCIAL PHOBIA**						
A: A marked and persistent fear of one or more social or performance situations	95	13	36	72.4	0.25^**^	
	29	15	(23.7)		(0.08–0.41)	0.32
B: Exposure to the feared social situation almost invariably provokes anxiety, which may take the form of a panic attack	93	10	39.5	73.2	0.32^***^	
	31	19	(25.9)		(0.16–0.47)	0.41
C: The person recognizes that the fear is excessive or unreasonable. In children, this feature may be absent	104	17	21	81.2	0.26^***^	
	9	8	(15.2)		(0.05–0.46)	0.40
D: The phobic situation is avoided or else endured with intense anxiety or distress	92	10	41	75.0	0.38^***^	
	28	22	(27.0)		(0.22–0.53)	0.46
E: Symptoms interfere with the person's normal routine, or cause marked distress	100	8	31.5	74.2	0.24^**^	
	31	12	(20.9)		(0.08–0.41)	0.37
F: Duration of at least 6 months in individuals under 18 years	85	13	44.5	70.4	0.30^***^	
	32	22	(29.3)		(0.15–0.45)	0.36
G: Symptoms are not due to the direct physiological effects of a substance or a medical condition	86	14	44.5	70.6	0.30^***^	
	31	22	(29.1)		(0.15–0.47)	0.35
H: Symptoms are unrelated to or another mental disorder or medical condition	87	13	44	71.2	0.31^***^	
	31	22	(28.8)		(0.15–0.47)	0.37

*Sample size for diagnostic categories vary due to missing values. Kappa coefficients are provided for diagnoses with a base rate higher than 10%. Kappa coefficients are not calculated if no disorder is identified by at least one informant. Yule's Y coefficients are incalculable if either cell frequency of the contingency tables equals zero. Significance of the kappa coefficients was determined with x^2^-exact tests*.

^*^ p < 0.05;

** p < 0.01;

*** *p < 0.001*.

On the level of current and lifetime diagnostic criteria (see Table 2), kappa values were significant and moderate for SAD (*k* = 0.53–0.67; *k* = 0.49–0.57), low for SoP (*k* = 0.22–0.38; *k* = 0.24–0.38), and low for SP (*k* = 0.27–0.63; *k* = 0.24–0.41). Again, parents indicated more criteria as positive than their children when large discrepancies were found. The criterion with the lowest agreement in SoP and SP was “symptoms interfere with the person's normal routine, or cause marked distress” (current: *k* = 0.22, lifetime: *k* = 0.24).

### Predictors of parent-child agreement

For the regression analyses, 85 parent-child dyads with complete interview data for current diagnostic criteria were available. The interview data conducted with mothers and both parents were calculated in one group because there was no significant difference between the number of current diagnostic criteria children and parents/mothers disagreed upon, *t*_(83)_ = 0.78, *p* = 0.44.

#### Child related predictors

Bivariate analysis (Pearson's *r*, two-tailed) showed no significant correlation between parent-child disagreement and the child's gender *r*_(85)_ = 0.006, *p* = 0.95, age, *r*_(85)_ = –0.19, *p* = 0.09 and total time of interaction, *r*_(67)_ = –0.129, *p* = 0.30.

#### Parent- and relation-related predictors

Overall, observed parental psychopathology was in a non-clinical range. The BDI and BAI scores were below the cut-off for mild symptoms. ASI and PSOC scores were at the lower bound of normative sample means reported for the general population (Johnston and Mash, 1989; Peterson and Plehn, 1999).

Bivariate analysis was calculated between parent-related factors and the number of disagreement. The analysis revealed that maternal BDI scores, *r*_(79)_ = 0.309, *p* = 0.01 correlated significantly to the number of disagreement. Maternal ASI scores, *r*_(80)_ = 0.190, *p* = 0.09 correlated not significantly to the number of disagreement. Social support rated by mothers *r*_(81)_ = −0.268, *p* = 0.03 and by fathers *r*_(66)_ = −0.219, *p* = 0.05 correlated significantly, indicating that higher maternal depression and lower social support reported by mother and father were associated with lower parent-child agreement. No significant correlations were found for maternal and paternal PSOC, BAI and paternal BDI and ASI scores, (*r* < 0.06, all *p* > 0.09).

Based on these results, four multiple regression analyses were performed with the frequency of disagreement on the diagnostic criteria as the dependent variable and (1) maternal BDI and ASI scores, (2) social support by mothers and fathers, (3) maternal BDI and social support scores, and (4) maternal BDI and paternal social support scores as independent variables. Beta coefficients are listed in Table 3, first line. The first model was significant, *F*_(2, 76)_ = 4.75, *p* = 0.011, explaining 11.1% of the variance. Maternal BDI scores significantly increased with the number of disagreements whereas ASI scores added no significant variance to the model after controlling for depression scores. The second model was significant, *F*_(2, 62)_ = 3.89, *p* = 0.028 explaining 11.1% of the variance. The predicting value of social support reported by mothers and fathers did not remain significant after controlling for each other. The third model was significant, *F*_(2, 76)_ = 4.55, *p* = 0.014 explaining 10.7% of the variance. Maternal social support added no significant variance to the model after controlling for depression scores. The fourth model was significant, *F*_(2, 60)_ = 7.25, *p* = 0.002 explaining 19.5% of the variance. Higher maternal depression and lower social support reported by the father predicted therefore parent-child disagreement.

**Table 3:** **Multiple regression analyses of the total number of parent-child disagreement on current diagnostic criteria and maternal depression (BDI), maternal anxiety sensitivity (ASI), and parental social support**.

		***R^2^***	**Beta**
Model 1	Depression (BDI) mother	0.111	0.285^*^
	Anxiety (ASI) mother		0.128
Model 2	Social support mother	0.111	−0.219
	Social support father		−0.180
Model 3	Depression (BDI) Mother	0.107	0.256^*^
	Social support mother		−0.120
Model 4	Depression (BDI) mother	0.195	0.346^**^
	Social support father		−0.250^*^

* p < 0.05;

** *p < 0.01*.

## Discussion

The present study adds to previous research by examining parent–child agreement on all three diagnostic levels in one study design focusing on anxiety disorders as the most prevalent disorder in childhood. These diagnostic levels were (a) diagnostic category (anxiety disorder), (b) specific anxiety diagnoses (SAD, SoP, SP), and (c) diagnostic criteria (e.g., criterion A of social anxiety disorder). Furthermore, the present study adds to previous research by providing interview data of mothers and both parents from children between 7 and 18 years old representing the diversity in the clinical field. The use of a clinical and a non-clinical sample enhanced moreover the generalization of the results.

### Parent-child agreement on diagnostic category and diagnoses

In accordance with earlier studies (e.g., Grills and Ollendick, 2003; Comer and Kendall, 2004; Weems et al., 2011; Propp et al., 2014), parent-child agreement was low on the diagnostic category (average *k* = 0.22), higher but still low on specific anxiety diagnoses (average *k* = 0.34) and moderate on diagnostic criteria (average *k* = 0.52). A possible explanation for this difference might be that important diagnostic information is assessed at the level of diagnostic criteria that might get lost in the course of the diagnostic evaluation. For example, a parent-child dyad would agree on the level of criteria but not on the level of diagnoses and diagnostic category if parent and child agree on criterion A of SAD (e.g., child is extremely afraid of being separated from the parents) but not on criterion D of SAD (e.g., distressed or impaired in daily functioning). These findings highlight the importance of a combination of categorical and dimensional diagnostics since subclinical problems might be identified at the level of criteria but remain undiscovered at the level of diagnoses.

### Parent-child agreement on diagnostic criteria

As expected, parent-child agreement on the level of diagnostic criteria varied between the types of anxiety disorder. Parents and children agreed more on criteria of SAD compared to SoP and SP replicating results of earlier studies (Jensen et al., 1999; Choudhury et al., 2003; Grills and Ollendick, 2003). Agreement on SAD was probably higher in the present sample because symptoms of SAD are separation-related (e.g., clinging, avoidance of being alone) and therefore more easily noticeable for parents than symptoms of SoP or SP. Furthermore, the prevalence rate for SAD was higher (*n* = 69) than for SoP (*n* = 42) and SP (*n* = 49) and therefore less susceptible for bias.

In general, parents endorsed more criteria than their children across all anxiety disorders analyzed in this study. One example therefore is the criterion E (“symptoms interfere with the person's normal routine, or cause marked distress”) of current and lifetime SP and SoP. These findings are supported by earlier studies that found that parents report generally more symptoms than their children (MacLeod et al., 1999; Martin et al., 2004). Anxious children, especially those with social phobia were identified to report fewer symptoms, particularly social avoidance than their parents (DiBartolo et al., 1998). A possible explanation might be that anxious children neglect symptoms to avoid a possible negative evaluation by seeming non-distressed. Alternatively, anxious children avoid negative feelings that might come up when they are reminded of anxiety provoking situations. Additionally, psychotherapeutic appointments are usually initiated by parents and not by children themselves. Parents might therefore have a clearer idea of concerned problems and why they are seeking help than their children which might result in lower diagnostic agreement.

### Predictors on the number of anxiety symptoms parent and child agree on

The predictive analyses showed that the child's age and gender predicted not significantly the number of parent-child agreement. In spite of mixed results, the present findings replicated the results of the majority of empirical work that showed no effect of gender or age (e.g., Grills and Ollendick, 2003; Propp et al., 2014). The inconsistent findings in the existing literature might be caused by different age groups and age ranges covered in earlier studies. Some studies, for example, covered younger children (8–12, Barbosa et al., 2002; 7–14, Comer and Kendall, 2004), older children (11–17, Propp et al., 2014) or wider age ranges (7–18, Brown-Jacobsen et al., 2011; 7–17, Hoffman and Chu, 2015) with a mean age of around 11 years.

### Parental depression and anxiety

In accordance with Propp et al. (2014), maternal depression predicted parent-child agreement on diagnostic criteria of anxiety disorders. The analysis of social support indicated a correlational result. Lower social support was associated with lower parent-child agreement. However, this relation was not significant after controlling for maternal depression. It can therefore be assumed that maternal depression is a strong predictor of parent-child agreement. It is still open to discussion whether depressed parents report more accurately or are biased about the symptoms of their children (Richters, 1992; Moore and Fresco, 2012). However, clinical suggestions about the relation between maternal psychopathology and parent-child agreement can be provided. It can be expected that parent-child agreement on anxiety symptoms decrease when mothers report (1) depressive mood and (2) low levels of social support. Furthermore, a potential buffering effect of social support on maternal depression can be suggested due to the correlational relation between these predictors leading to higher levels of parent-child agreement. This idea is supported by earlier findings that social support is a moderator between maternal depression and behavioral problems in children (Lee et al., 2006).

### Social support

We were able to show for the first time that, analogue to mothers, paternal lower social support predicted lower parent-child agreement. No significant correlations were found for parental sense of competence, anxiety (sensitivity), and depression. So far, the impact of paternal variables on parent-child agreement is less studied than maternal variables (Grills and Ollendick, 2003). It has been suggested that mothers spend more time with their children and provide therefore more information about the child's behavior (De Los Reyes and Kazdin, 2004). This might be an explanation why fathers' psychopathology did not predict parent-child agreement in the present study. However, the positive correlation between reported social support of mothers and fathers indicated that this might be a shared predictor for parent-child agreement. Other than psychopathological variables, social support seemed to affect the whole family leading to a lower parent-child agreement.

### Parental sense of competence

Furthermore, contrary to our expectations, parental sense of competence had no predicting value for parent-child agreement in the present study. An association between parental depression, self-efficacy, and social support has been reported by different studies (Herwig et al., 2004; Lee et al., 2006). In view of the high predicting value of maternal depression on parent-child agreement, it could be expected that mothers with a high level of parental self-efficacy would show higher levels of parent-child agreement due to increased parental capacity including responsive caretaking and more attention to the child's signals (Coleman and Karraker, 2000). This hypothesis was supported in the present study. Additionally, no significant effect was found for the amount of time parents and children spent together. A possible explanation might be that not the total amount of time but the quality of parent-child interaction predicts the extent to which parents and children agree upon behavioral and emotional difficulties (Grills and Ollendick, 2002). A further investigation of a potential moderating effect of different aspects of parenting (e.g., quality of communication) might be of value.

### Limitations

Despite the high number of participants, not all anxiety disorders (e.g., panic disorder, GAD) included in the structured diagnostic interview were represented because of the low base rates in this age group. Low base rates can lead to a limited interpretation of kappa because of its base rate dependency. Either a very low or a very high prevalence of the observed event leads to an underestimation of the kappa coefficient (Cicchetti and Feinstein, 1990; Sim and Wright, 2005). Another limitation is the limited generalizability of the present finding regarding parent- and child related predictors. Because of feasibility reasons we were only able to examine this research question in the sample of children with anxiety disorders treated in the university-related outpatient clinic. These results might therefore not be generalizable to inpatient clinical samples due to evidence that parent-child agreement differs between inpatient and outpatient clinical settings (e.g., Martin et al., 2004; van der Ende and Verhulst, 2005).

In conclusion, based on the existing research on parent-child agreement in structured diagnostic interviews, parent-child agreement in anxiety disorders can be expected to be low to moderate as replicated in the present study. Additionally, the present findings indicated that diagnostic agreement varies (1) between the diagnostic levels and (2) as a function of type of anxiety disorder. Higher parent-child agreement can be expected if the diagnostic symptoms are assessed on the level of diagnostic criteria rather than on diagnoses. Clinicians might benefit from a closer look at the anxiety symptoms parents and children agree upon in respect to diagnostic and therapeutic processes (Achenbach et al., 1987; Reyes, 2011).

There is additional growing evidence that parental psychopathology, and more specifically maternal depressive mood decreases the level of parent-child agreement. Until now, it is an open question whether maternal psychopathology increases the validity of mothers' diagnostic report. Additionally, low social support of both parents was associated with lower parent-child agreement. The assessment of maternal psychopathology and familial social support might be of additional value in interpreting diagnostic parent-child agreement.

## Ethics statement

Our treatment of human subjects and the conduct of research complied with the ethical standards set forth by APA and was approved by the local ethical committee for medical research. All subjects gave written informed consent in accordance with the Declaration of Helsinki.

## Clinical trials

Registration identification number: NCT00255112. Registry URL: http://www.clinicaltrials.gov

## Author contributions

LP analyzed the data and drafted the manuscript. MN, TI, and SM conducted the research. SS and TI designed the research, and provided critical feedback. All authors read and approved the final manuscript.

## Funding

This study was partly supported by Swiss National Science Foundation Grant 105314-116517, “Etiology and Psychological Treatment of Separation anxiety disorder-specific cognitive-behavioral therapy (CBT) program for children with separation anxiety disorder (SAD)” awarded to SS.

### Conflict of interest statement

The authors declare that the research was conducted in the absence of any commercial or financial relationships that could be construed as a potential conflict of interest.

## Abbreviations

SAD: Separation anxiety disorder
SoP: Social phobia
SP: Specific phobia
Kinder-DIPS: Structured Diagnostic Interview for Mental Disorders in Children (“Diagnostisches Interview bei psychischen Störungen im Kindes- und Jugendalter”).
