# Supplementary material for: Parent-Child Diagnostic Agreement on Anxiety Symptoms with a Structured Diagnostic Interview for Mental Disorders in Children
Source: Front Psychol. 2017 Mar 27;8:404. doi: 10.3389/fpsyg.2017.00404 (PMC5366335; doi:10.3389/fpsyg.2017.00404)
Supplement: Supplementary file 1 [file Table1.DOCX]

|  |  | *N* (%) | *M*  (*SD*) |  |
| --- | --- | --- | --- | --- |
|  | Children |  |  |  |
|  | Age (years) |  | 10.94 (2.22) |  |
|  | Gender:  Male  Female | 80 (48.2)  86 (52.8) |  |  |
|  | Parents |  |  |  |
|  | Age of mother (years) |  | 41.81 (4.84) |  |
|  | Age of father (years) |  | 45.13 (6.89) |  |
|  | Marital status  Married / partnership  Single  Separated / divorced  Widowed  Missing | 127 (76.5)  6 (3.6)  23 (13.9)  2 (1.2)  8 (4.8) |  |  |
|  | Occupation of mother:  Homemaker  Manual worker  Employee  Public servant  Self-employee  Out of work  Missing | 54 (32.5)  1 (.6)  76 (45.8)  6 (3.6)  23 (13.9)  0  6 (4.8) |  |  |
|  | Occupation of father:  Homemaker  Manual worker  Employee  Public servant  Self-employee  Out of work  Missing | 1 (.6)  10 (6.0)  96 (57.8)  9 (5.4)  26 (15.7)  1 (.6)  23 (15.1) |  |  |
|  | Family characteristics: |  |  |  |
|  | Child lives with  Biological parents  Adoptive parents  Single mother  Biological mother and partner  Missing | 139 (83.7)  0  9 (5.4)  7 (4.2)  11 (6.6) |  |  |
|  | Number of siblings |  | 1.23 (0.91) |  |

Appendix A

*Sociodemografic data of the final sample (N = 166)*
